# Identification of tag single-nucleotide polymorphisms in regions with varying linkage disequilibrium

**DOI:** 10.1186/1471-2156-6-S1-S73

**Published:** 2005-12-30

**Authors:** Priya Duggal, Elizabeth M Gillanders, Rasika A Mathias, Grace P Ibay, Alison P Klein, Agnes B Baffoe-Bonnie, Liang Ou, Ian P Dusenberry, Ya-Yu Tsai, Peter S Chines, Betty Q Doan, Joan E Bailey-Wilson

**Affiliations:** 1 Inherited Disease Research Branch, NHGRI/NIH, Baltimore, MD, USA; 2 Fox Chase Cancer Center, Division of Population Science, Philadelphia, PA, USA; 3 CIDR, Johns Hopkins Medical School, Baltimore, MD, USA; 4 Genome Technology Branch, NHGRI/NIH, Bethesda, MD, USA

## Abstract

We compared seven different tagging single-nucleotide polymorphism (SNP) programs in 10 regions with varied amounts of linkage disequilibrium (LD) and physical distance. We used the Collaborative Studies on the Genetics of Alcoholism dataset, part of the Genetic Analysis Workshop 14. We show that in regions with moderate to strong LD these programs are relatively consistent, despite different parameters and methods. In addition, we compared the selected SNPs in a multipoint linkage analysis for one region with strong LD. As the number of selected SNPs increased, the LOD score, mean information content, and type I error also increased.

## Background

A variety of methods to identify haplotype tagging single-nucleotide polymorphisms (ht-SNPs) and tagging SNPS are currently available. These programs employ different algorithms or methods to identify a SNP, which may include the identification of haplotypes, haplotype blocks, and regions of linkage disequilibrium (LD). However, a comprehensive comparison of these programs is lacking. We examined several different tagging SNP selection programs using the Collaborative Studies on the Genetics of Alcoholism (COGA) dataset while altering the amounts of LD (moderate to complete LD), the physical distance of the regions considered (68 kb-435 kb), and the haplotype or minor allele frequency. For each program, we present a comparison of the number of tagging SNPs selected in 10 regions on 9 chromosomes and the percentage of agreement among the programs. Additionally, we examined the effects of selected tagging SNPs on multipoint linkage analysis. Dense SNP panels are likely to result in increased inter-marker LD, which violates the assumption of equilibrium of markers in multipoint linkage analysis. We examined the results of multipoint linkage analysis using all the SNPs in a region with LD and only those tagging SNPs selected by the different programs.

## Methods

### Population and haplotype reconstruction

COGA is a 6-center collaborative study designed to identify loci for alcoholism and related disorders and these data were available as part of the Genetic Analysis Workshop 14 (GAW14) [1]. We restricted our analysis to one ethnicity to limit bias on allele frequencies, LD measurements, and haplotype reconstruction. All individuals classified as White, non-Hispanic (*n *= 1,074) were included. There were 102 pedigrees with a mean size of 10.5 (SD ± 5.1) and 332 founders. From the total founders we randomly ascertained one founder per pedigree (*n *= 102). Then, we randomly ascertained 30 founders who were used for all subsequent analyses. Since some tag SNP programs require haplotypes we used an expectation maximization (EM) algorithm as implemented in the program SNPHAP (v1.1) [2] to reconstruct phase unknown haplotypes from the 30 founder individuals. For each individual we used the haplotypes with the highest probability.

### Physical distance map

Because the physical positions of SNPs from Illumina and Affymetrix were based on different assemblies of the human genome, we obtained updated physical locations for each SNP from dbSNP on NCBI Build 34 to generate an integrated, high-density map. For SNPs with multiple physical locations, we chose the position closest to the previous build. SNPs without physical positions were excluded (*n *= 322). The Illumina map (4,720 SNPs) and the Affymetrix map (10,798 SNPs) were then merged. This merged SNP map was used so that we would have definite regions of LD due to increased SNP density. There were 94 SNPs common to both maps. Genotyping data from Illumina for these 94 SNPs were used due to the lower overall missing rate (Illumina = 0.05%, Affymetrix = 5.25%). In total we had 15,424 SNPs across the whole genome.

### SNP selection programs

We used 7 different SNP selection programs and then compared the overall percentage of agreement between the programs for the selected tag SNPs in 10 regions. These methods are very complex and each method cannot be fully explained here, but we encourage the reader to consult the referenced papers. We provide details of how we ran each program since there are many options in each program.

SNPTagger[3] uses previously inferred haplotypes, which are sorted in descending order according to their frequencies (frequencies ≥1% are reported). Then all markers are ranked according to their diversity values in the included haplotypes, calculated by counting the number of major and minor allele appearances in each column/marker separately, and choosing whichever is smaller[4].

Tag SNP[5] proposes a multi-step EM algorithm begins with the calculation of the haplotype dosage, δ_h_(H), the count of the number of copies of a specific haplotype *h *(0, 1 or 2) contained in the true pair of haplotypes for each individual conditional on the individual's genotype data, and over all ordered haplotype pairs. Selecting subsets of SNPs, the squared correlation between the true and predicted haplotype dosage (*R*^2^*h*) is calculated. The lowest haplotype frequency was set to 0.1%, and the set of SNPs above which the addition of any further SNPs did not yield an improved *R*^2^*h *were selected.

Chapman/HTSNP is a set of programs)[6,7] that can be run within the statistical software STATA (v8) to identify a minimal set of tag SNPs using different criteria including percent diversity explained (PDE) and *R*^2^. PDE is an index that measures the total haplotype diversity if only the htSNPs are used. *R*^2 ^is a variant of the coeffiecient of determination, that is the percentage of variance explained by regression [7]. We used the exhaustive search algorithm htsearch to find the minimal set of tag SNPs that both maximized the percent diversity (PDE > 0.98) and the *R*^2 ^(> 0.98). All analyses used a minor allele frequency (MAF) > 0.1%.

Nyholt's[8] method uses spectral decomposition, SpD. The eigenvalues (λ) measure the variance of each SNP-SNP correlation, and the higher the correlation among the set of SNPs the greater the λ values. The program examines the factor loadings for each eigenvalue to determine which SNP captures the best information for each set of SNPs using an orthogonal rotation. The Meff option identifies the minimum subset of SNPs, which maximize the information of the SNP group.

The Meng method [9] uses a SpD matrix of pair-wise LD (*R*^2^) by calculating the eigenvalues, and applies a varimax-rotation procedure to the original set of eigenvectors. The rotation allows for an orthogonal transformation, thereby calculating the influence of each SNP on the eigenvector. To determine the number of the most influential SNPs contributing to the region, the proportion of variance explained was set to 90%. This high proportion was selected because the typical number of founders used is lower than those suggested by the authors. We implemented this method in the statistical program R.

We used HAPLOVIEW v2.05 [10] to create blocks utilizing the Gabriel et al. algorithm [11]. This algorithm uses the 95% confidence intervals (CIs) of pair-wise D' values to designate 2 SNPs as being in strong LD. The CI minima for 2 SNPs in strong LD are 0.98 (upper) and 0.70 (lower). A block is defined as a region over which 95% of informative comparisons are in strong LD. All markers with MAF < 5% were excluded and the minimum haplotype frequency was set to 0.1%. An accelerated EM algorithm, similar to Qin et al. [12] estimates haplotypes. Then all within-block SNPs are ranked in order of genotyping success rates and those SNPs that capture all haplotypes within a block are chosen as htSNPs.

We used HaploBlock Finder (v0.7) which utilizes the haplotype block definition proposed by Patil et al. [13] and the dynamic programming algorithm by Zhang et al. [14] to find the optimal block partition and tagSNPs. Using a set α, a block is defined if at least α percent of haplotypes are represented more than one time [13,14]. For this analysis we report the results from 95% chromosomal coverage, MAF > 0.1%, and the default of 0.90 for htSNP coverage.

### Linkage analysis

**Figure 1:**
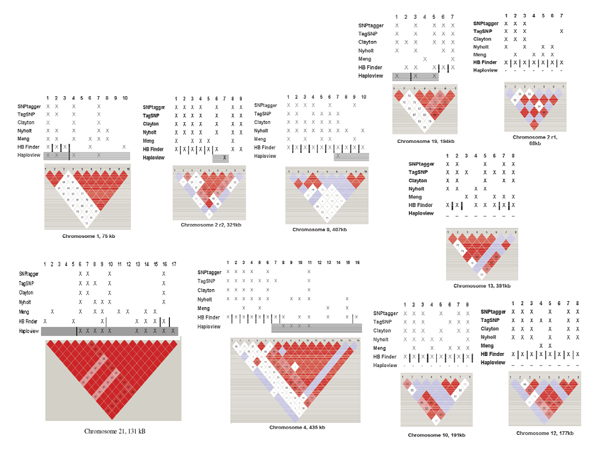
**Selected SNPs and linkage disequilibrium plots**. Figure 1 depicts the LD (D') and the tag SNPs selected by each program. Red regions depict D' = 1.0 with strong confidence. Light blue regions are D' = 1.0 but decreased confidence. White regions are D' < 1 and state D' within the box. An X denotes that SNP was selected by the program. The gray shading in the HAPLOVIEW row represents the Gabriel blocks. The dark lines represent breaks between blocks for both HAPLOVIEW and HaploBlock Finder. For some chromosomes no blocks were identified and this is indicated by hatch marks across the SNPs.

We performed multipoint linkage analysis with MERLIN [15] on the chromosome 21 region (Figure 1) using the different SNPs selected by the programs. We used the *pedwipe *command to eliminate any marker inconsistencies, and some pedigrees were split into smaller pedigrees because of size/marker constraints of the program. For affection status we used a marker as a dominant trait, by selecting a SNP (rs2835626, SNP 7), from the 17 SNPs on our chromosome 21 region and coding the minor allele as diseased (allele frequency 0.30). This "trait" SNP was selected by allele frequency without reference to the tag SNPs selected by each program and it was possible for the SNP "trait" marker to be excluded from the set of tag SNPs by any program. In addition, we used another marker, tsc0041859, as a dominant trait caused by an "unlinked" locus on chromosome 20, to determine if there was a large increase in type I error.

## Results

Figure 1 depicts LD, D', (from HAPLOVIEW) and the tagging SNPs selected by each program. Across the various programs there was some consistency in the identification of SNPs for these 10 regions. However, Meng had a consistently lower percentage of agreement than the other programs (Table 1). In addition to a possible program implementation error, this may be explained by the high LD in certain regions. Meng et al. suggest using a lower "variation explained" when the LD is high, however in order to achieve consistency across the regions/programs we used a set 90%. Thus in regions of high LD, more markers are retained than other programs. Additionally, Meng is optimal for a sample size of 50–100 individuals; however we used 30 founders because of processing limitations in the construction of haplotypes for other programs. The two block method programs, HaploBlock Finder and HAPLOVIEW, *appeared *to be inconsistent in the selection of SNPs with a minimal percent agreement of only 26%. However, HAPLOVIEW using Gabriel's block definition was very stringent so that many regions did not qualify for SNP selection despite LD among SNPs since no blocks were identified. In contrast, HaploBlock Finder forces single SNPs to have individual blocks and at least one SNP per block must be chosen. If we required HAPLOVIEW to also choose all the SNPs outside of the Gabriel-defined blocks, the percentage of agreement between the 2 programs was drastically increased to 77%. The diversity or *R*^2^-based programs, SNPtagger, TagSNP and htSNP, all performed similarly across the regions with 87–95% agreement. This is encouraging because not all of the programs have the same requirements although they use a comparable measurement.

The results of our multipoint linkage analysis (Table 2) suggest that in a region of strong LD, not all of the SNPs are necessary to garner a strong linkage signal. However, the additional SNPs do increase the power and the strength of the LOD score, with a range of 13.50 (17 SNPs) to 11.44 (4 SNPs), decreasing as the number of SNPs decreased. In some cases the simulated trait-SNP was included as a marker because it had been selected by the program, however even when the simulated trait-SNP was excluded as a marker, the LOD scores remained high, presumably because of the strong LD and density of the other SNPs in the region. The mean information content also increased marginally as the number of SNPs increased, with a range of 0.58 (4 SNPs) to 0.69 (17 SNPs). In this region of strong LD, as more SNPs were included in the analysis, the type I error appeared to increase from 4 SNPs (*p *= 0.4) to 17 SNPs (*p *= 0.02), although only to a nominal level for the simulated trait of an unlinked region.

## Conclusion

We performed a comprehensive comparison of different tagging SNP programs to determine if the amount of LD or the size of the region influenced the selection of tagging SNPs. Overall, HaplotypeBlock Finder and the SpD method tend to choose more SNPs, and that the diversity or *R*^2 ^measurements were more likely to choose fewer SNPs. However, there was consistency among the programs and it suggests that in regions with moderate to complete LD these programs perform similarly despite different parameters and/or algorithms. Additionally, all of the tag SNPs performed well for our multipoint linkage analysis of a single region on chromosome 21 despite vastly different numbers of total SNPs used. This region had very strong LD and although each of the programs reduced the number of SNPs in the region by picking a subset to be tag SNPs, there was still residual LD among the SNPs selected, which would still violate the assumption of linkage equilibrium between markers in multipoint linkage analysis. Therefore, we do not suggest using only these methods as a measure to remove LD prior to linkage analysis. Although it is difficult to reach a conclusion from one replicate, our study suggests that increased SNP density may improve the power to detect linkage but also may increase the associated type I error.

## Abbreviations

CI: Confidence interval

COGA: Collaborative Studies on the Genetics of Alcoholism

EM: Expectation maximization

GAW14: Genetic Analysis Workshop 14

LD: Linkage disequilibrium

MAF: Minor allele frequency

PDE: Percent diversity explained

SNP: Single-nucleotide polymorphism

SpD: Spectral decomposition

## Figures and Tables

**Table 1:** The percent agreement between selected SNPs across tag-SNP programs.

	**SNPtagger**	**TagSNP**	**HTSNP**	Nyholt	Meng	HB Finder
**TagSNP**^**a**^	87					
**HTSNP**	95	88				
Nyholt^b^	77	74	78			
Meng	46	47	43	53		
*HB Finder*^*c*^	62	65	63	65	46	
*Haploview*	48 (67)^d^	45 (74)	47 (68)	47 (68)	54 (45)	26 (77)

There are 100 SNPs in 10 regions.

^a^**Bold**, *R*^2 ^or percent diversity based programs.

^b^Standard, spectral decomposition programs.

^c^*Italics*, block method programs.

^d^Parenthesis indicates the percent agreement when SNPs chosen by HAPLOVIEW included those outside of the Gabriel blocks.

**Table 2:** Multipoint linkage analysis ordered by number of tagSNPs for the chromosome 21 region.

	# of SNPs	LOD score (*p*-value) simulated trait (chromosome 21) rs2835626	LOD Score (*p*-value) simulated trait (chromosome 20) tsc0041859	Mean information content
**SNPtagger/HTSNP**^**a**^	4	11.44 (<0.00001)	0.03 (0.4)	0.58
Nyholt	5	11.70 (<0.00001)	0.17 (0.2)	0.60
**TagSNP**	5	12.03 (<0.00001)	0.15 (0.2)	0.60
**Haploview**	6	12.42(<0.00001)	0.30 (0.12)	0.62
HB Finder	7	12.63 (<0.00001)	0.80 (0.03)	0.61
Meng^b^	8	13.27 (<0.00001)	1.08 (0.013)	0.60
All SNPs	17	13.50(<0.00001)	0.86 (0.02)	0.69

^a^**Bold**, program included the simulated trait on chromosome 21.

^b^Black, program _______________________________.
